# Congenital absence of the gallbladder in a child: a case report

**DOI:** 10.3389/fped.2024.1440383

**Published:** 2024-07-26

**Authors:** Xiao Wei, Liying You, Chun Liu, Xu Xu

**Affiliations:** Department of General Medicine of Ganmei Hospital, Affiliated to Kunming Medical University (Kunming First People’s Hospital), Kunming, Yunnan, China

**Keywords:** congenital absence of the gallbladder, hepatic ultrasound, liver function abnormalities, fatty liver, case report

## Abstract

**Background:**

Congenital absence of the gallbladder (CAGB) is an exceedingly rare embryological anomaly of the biliary system, with a complex etiology involving the failure of gallbladder formation during embryogenesis. Clinical manifestations are diverse; most patients are asymptomatic, while some present with symptoms such as biliary colic. The complexity of its clinical presentation and radiological features renders diagnosis challenging.

**Case presentation:**

Fetal ultrasound at 22 weeks of gestation revealed an absent gallbladder. At 9 years and 11 months of age, the child exhibited significant weight gain and abnormalities. Abdominal ultrasound and magnetic resonance images demonstrated fatty liver and gallbladder agenesis. Liver function tests indicated mild abnormalities, with aspartate aminotransferase at 67 IU/L and alanine aminotransferase at 44 IU/L. Following 6 months of hepatoprotective and lipid-lowering therapy, a satisfactory treatment response was achieved, with normalization of liver function and improvement in fatty liver.

**Conclusions:**

CAGB may be associated with other congenital abnormalities, although isolated cases are uncommon. Clinically, it may manifest as nonspecific biliary, gastrointestinal, or urinary symptoms, mimicking various digestive disorders and leading to misdiagnosis. Genetic sequencing and in-depth embryological research may elucidate the etiology and enhance diagnostic accuracy.

## 1 Introduction

Congenital absence of the gallbladder (CAGB) is an exceptionally rare congenital biliary malformation, with a reported global incidence of 0.01%–0.065% (1). Since Lemery's initial description in 1701, approximately 500 cases have been documented worldwide (2). While most individuals with CAGB remain asymptomatic, some may exhibit signs and symptoms reminiscent of gallbladder disease. The pathogenesis of CAGB remains enigmatic, but it is presently regarded as a congenital malformation (3), potentially attributable to the failure of gallbladder bud formation or a deficiency in vacuolization during embryonic development.

CAGB can be classified into three categories: multiple congenital anomalies, asymptomatic, and symptomatic (4). Although some individuals with CAGB may remain symptom-free throughout life without associated complications, abdominal ultrasonography can definitively diagnose asymptomatic cases (5). Additionally, some patients' symptoms may resemble those of other digestive disorders, primarily presenting as upper right abdominal pain, gastrointestinal symptoms, biliary colic, and occasionally jaundice, with recurrent episodes (6, 7). These symptoms may be attributable to concomitant common bile duct stones and intrahepatic bile duct stones, or they may be caused by isolated CAGB, the reasons for which remain obscure, thus increasing the likelihood of misdiagnosis (8). Imaging studies may also have limitations, necessitating an enhanced understanding of this condition among surgeons and radiologists, who must synthesize clinical presentation and auxiliary examinations to make comprehensive judgments, thereby reducing misdiagnoses and unnecessary exploratory surgeries (9, 10).

We report a case of a male fetus diagnosed with CAGB during a prenatal checkup. The gallbladder was not visualized during the 22-week prenatal examination, and the fetus was born in March 2011, developing normally. In February 2022, at the age of 9 years and 11 months, the child underwent evaluation due to significant weight gain, and abdominal ultrasound and magnetic resonance images (MRI) revealed fatty liver and CAGB.

## 2 Case presentation

**Figure 1:**
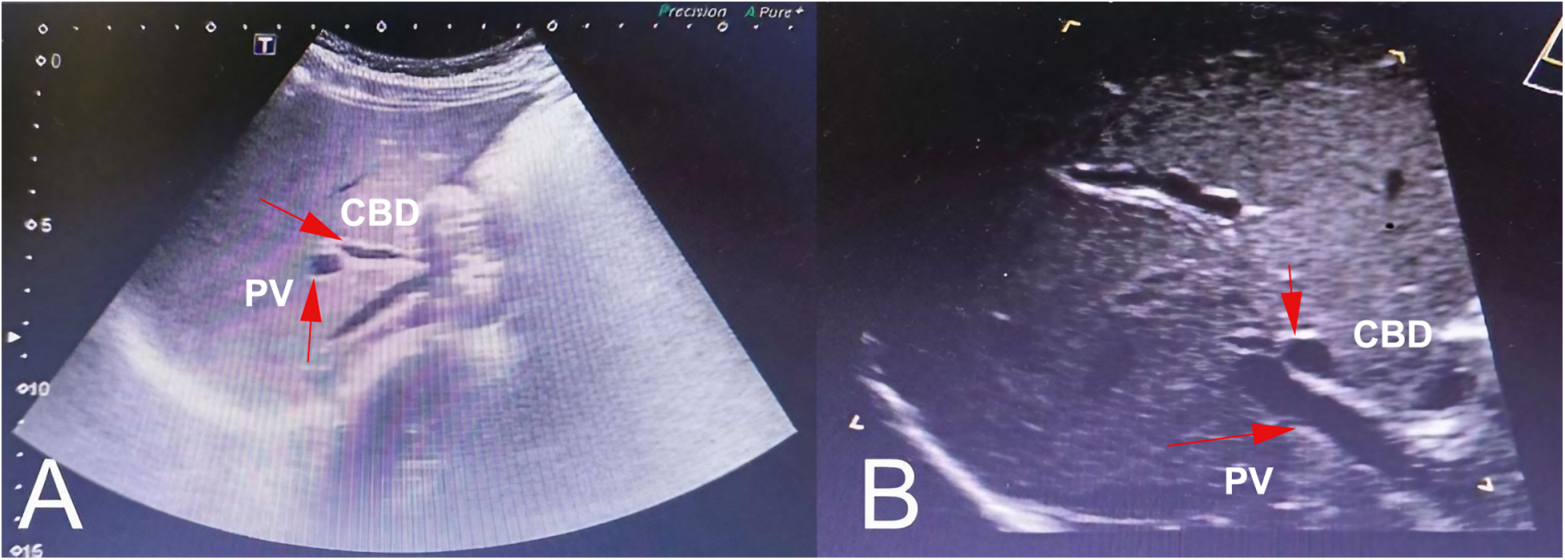
Ultrasound scans of a pediatric patient with CAGB. An ultrasonographic image (**A**) and after magnification (**B**) of the gallbladder showed a deficiency in the gallbladder. The common bile duct (CBD) and portal vein (PV) are indicated by arrows for reference. No gallbladder or gallbladder remnant was visible in the expected location.

A male patient, born at full term with a birth weight of 2,750 g, was diagnosed with CAGB during a prenatal ultrasound at 22 weeks of gestation. The patient's growth and development were normal until the age of 9 years and 11 months when he presented with significant weight gain. At presentation (10 years old), the patient weighed 47 kg with a height of 142 cm, resulting in a BMI of 23.3 kg/m^2^. His blood pressure and pulse rate were within normal limits, and his body temperature was 36.7°C. Liver function tests revealed mild abnormalities with aspartate aminotransferase (AST) at 67 IU/L and alanine aminotransferase (ALT) at 44 IU/L. These findings, along with the fatty liver observed on MRI, are likely associated with the patient's rapid weight gain rather than being a direct consequence of the congenital absence of the gallbladder. Importantly, the conjugated bilirubin level was within normal limits (0.2 mg/dl), which helped rule out biliary atresia, as elevated conjugated bilirubin is typically observed in that condition. Upper gastrointestinal endoscopy was not performed as the patient's symptoms and other diagnostic findings did not suggest acid peptic disease. Additionally, ultrasonography demonstrated an absent gallbladder (Figure 1A). At higher magnification (Figure 1B), the gallbladder absence was more apparent. This finding was consistent with the diagnosis of congenital absence of the gallbladder. In clinical practice, the absence of the gallbladder on ultrasound typically necessitates further diagnostic imaging or tests to confirm the diagnosis and exclude other conditions.

**Figure 2:**
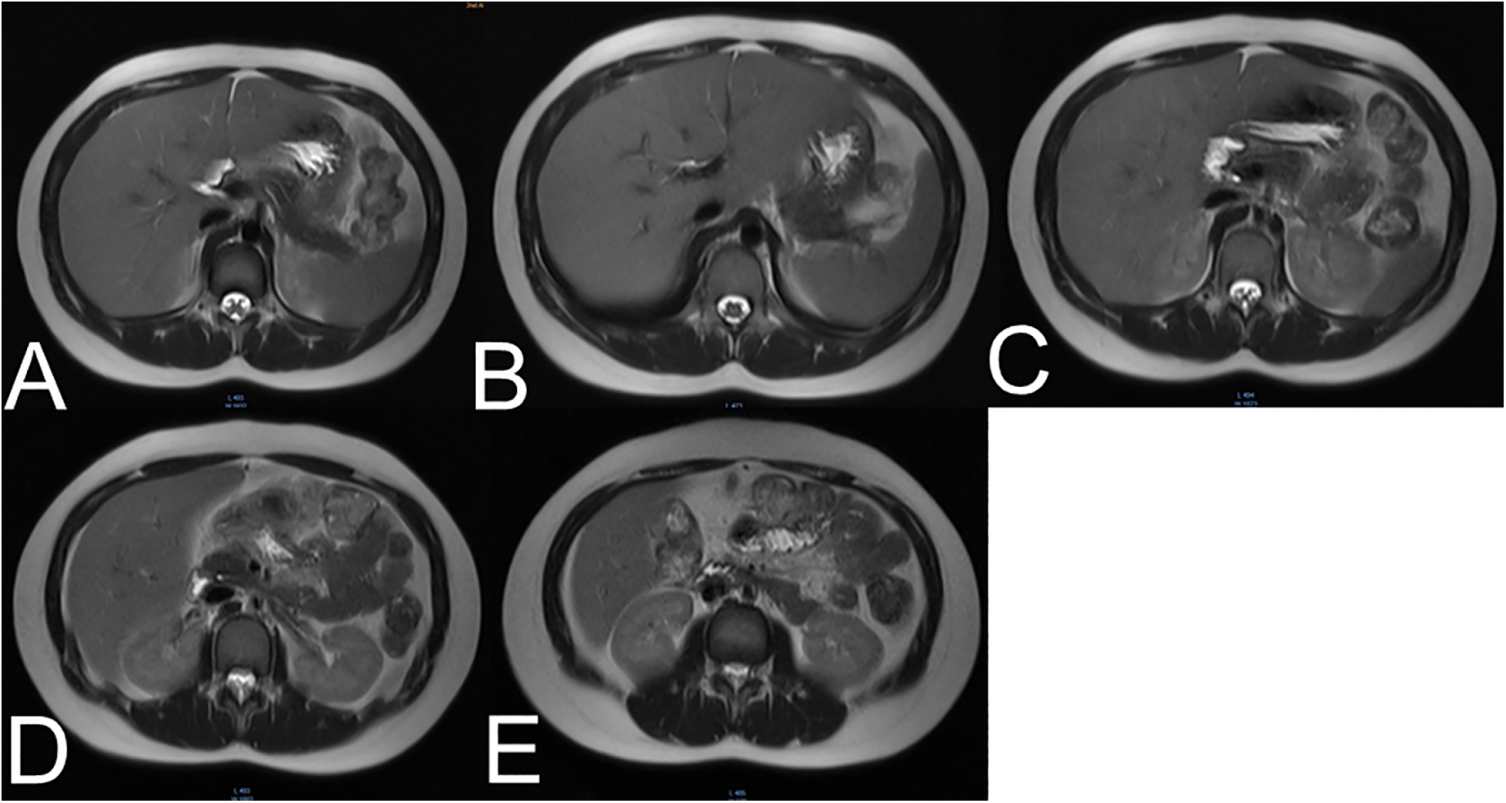
(**A–E**) Five different cross-sectional MRI scans of the abdomen. These findings usually illustrate various segments of the liver and surrounding anatomical structures, which indicate mild hepatic steatosis.

MRI of the abdomen revealed a fatty liver with an absence of the gallbladder (Figure 2). No other bile duct variations were observed in imaging studies. The images clearly illustrated various segments of the liver and surrounding abdominal anatomy. The grayscale property of the images facilitated a detailed examination of internal structures, with varying shades representing different tissues and densities. The liver and its vascular structures were distinctly visible (Figure 2A). A similar section with more pronounced vascular features was subsequently observed (Figure 2B). Moreover, the MRI revealed an area of altered tissue density, associated with fatty liver disease (Figure 2C), and provided additional views of the liver and surrounding tissues, further corroborating the diagnosis of fatty liver (Figures 2D,E). Variations in tissue density within the images may indicate fat deposition in the liver, while the absence of certain expected anatomical features could suggest underdevelopment or absence of the gallbladder. Following six months of hepatoprotective and lipid-lowering treatment, liver function normalized, and fatty liver improved. The patient was satisfied with the diagnostic and the proposed care. The episode of care is organized as a timeline in Table 1.

**Table 1:** A timeline with relevant data from the episode of care.

Time point	Event description
Gestational week 22	Gallbladder not observed during prenatal checkup.
March 2011	The male fetus was born, and normal growth and development were observed.
February 2022	At 9 years and 11 months of age, the patient presented with significant weight gain. Abdominal ultrasound and abdominal magnetic resonance imaging revealed: fatty liver, agenesis of the gallbladder, and mild liver function abnormalities.
6 months later	After 6 months of liver protection and lipid-lowering treatment, liver function returned to normal, and fatty liver improved.
One year later	At follow-up, the patient continued in good clinical condition.

The patient and his family expressed satisfaction with the diagnostic process and the proposed care plan. They reported feeling relieved to have a clear explanation for the prenatal findings and were committed to following the recommended lifestyle modifications and treatments. The patient's growth and development were monitored every six months from age 10 to 13, with all measurements within the normal range. At age 13, the patient's height was 164.5 cm and weight was 59 kg, indicating normal growth progression.

## 3 Discussion

CAGB is an uncommon condition characterized by the failure of proper gallbladder development during fetal life (11). It is generally accepted that this anomaly is linked to the development of the hepatic diverticulum. During the fourth week of embryogenesis, the hepatic diverticulum arises from the endoderm at the junction of the foregut and yolk stalk, ultimately giving rise to the liver and gallbladder, with the cystic duct originating from its proximal portion (12). Patients with CAGB often experience vague symptoms such as pain in the upper right abdomen, nausea, or jaundice. Diagnosing CAGB can be challenging, as it may be mistaken for gallstones, occasionally leading to unnecessary surgeries (13).

The gallbladder is an organ that stores bile for digestion; its absence may affect digestive function (14). However, in isolated cases of gallbladder absence, no special treatment is generally required, and pregnancy can continue with regular postnatal observation (15). Non-visualization during prenatal screening, potentially due to technical issues or natural causes, necessitates a follow-up ultrasound to check for abnormalities. Gallbladder absence could indicate other anomalies and requires careful monitoring, as observed in a case at 22 weeks gestation. Furthermore, gallbladder absence may be associated with other anomalies, such as duodenal atresia or biliary atresia (16). In our case report, the gallbladder absence was noted during a 22-week gestational check-up, necessitating follow-up to ensure normal development. Moreover, if the child exhibits any abnormal symptoms during growth and development, medical treatment or other interventions may be warranted based on the specific situation. The concurrent occurrence of gallbladder agenesis and fatty liver in this young patient underscores the need for comprehensive evaluation and management of metabolic health, particularly in the context of rapid weight gain in pediatric patients. The concurrent occurrence of gallbladder agenesis and fatty liver in a young patient underscores the need for early lifestyle and medical interventions to prevent advanced liver disease, particularly in the context of pediatric patients.

In summary, the preoperative diagnosis of CAGB is challenging. During the history-taking process, it is essential to inquire about the patient's family history of CAGB or other related systemic variations (17). Imaging studies follow, with surgical exploration being the last resort. Clinically, ultrasound is often used to examine gallbladder diseases, with a sensitivity of over 95% (18). In our case, the initial ultrasound examination indicated that the gallbladder was not visualized within the gallbladder fossa. According to the proposed diagnostic imaging protocol for biliary diseases, patients suspected of having cholecystitis with unclear ultrasound images or apparent atrophy should undergo upper abdominal MRI, CT, or magnetic resonance cholangiopancreatography (MRCP) examination, which may reduce the misdiagnosis rate of CAGB (19).

As patients with CAGB grow, they may experience various long-term effects on their digestive system and metabolism. The absence of a gallbladder can lead to biliary dyskinesia, which is believed to be caused by increased tonicity of the sphincter of Oddi. This condition is comparable to postcholecystectomy syndrome and may explain the biliary colic experienced by some patients with CAGB. Interestingly, ursodeoxycholic acid has shown promise in relieving symptoms of biliary dyskinesia and may potentially have a role in managing CAGB-related symptoms (20).

While many individuals with CAGB remain asymptomatic throughout their lives, suggesting that the body can adapt to the absence of a gallbladder, others may present with various symptoms. According to previous studies, right upper quadrant pain is present in 90% of symptomatic cases, nausea and vomiting in 60%, and jaundice in 35% (21, 22). These symptoms can mimic other biliary pathologies, leading to potential misdiagnosis.

It's important to note that CAGB has been associated with malformations in other systems, particularly cardiovascular, skeletal, and abdominal wall abnormalities. There is also an association with trisomy 18 and Klippel-Feil syndrome (23). This underscores the importance of comprehensive evaluation and long-term follow-up for patients diagnosed with CAGB.

Regarding the risk of malignancy, while there is limited research due to the rarity of the condition, some studies have noted that the common duct is frequently found to be dilated at exploration, sometimes in the presence of stones. This anatomical alteration might potentially impact the long-term health of the biliary system, although more research is needed to establish any definitive link to increased cancer risk.

In this case, fatty liver and gallbladder absence were identified upon reevaluation of the gallbladder after MRI. We believe that patients with congenital gallbladder absence require a thorough assessment of their condition. It is also necessary to broaden the diagnostic approach, combining various examination methods, such as genetic sequencing and in-depth embryological research, to continuously improve diagnostic accuracy and avoid misdiagnosis.

It is important to note that the mild elevations in liver enzymes (AST 67 IU/L, ALT 44 IU/L) and the fatty liver changes observed on MRI are more likely associated with the patient's rapid weight gain rather than being a direct consequence of congenital absence of the gallbladder. Childhood obesity is a known risk factor for non-alcoholic fatty liver disease, which can lead to elevated liver enzymes and hepatic steatosis. This case highlights the importance of considering multiple factors when evaluating liver function abnormalities in pediatric patients, even in the presence of rare congenital anomalies like gallbladder agenesis.

## 4 Conclusion

CAGB is a rare embryological anomaly of the biliary system, presenting challenges in diagnosis and treatment. CAGB may be associated with other congenital abnormalities, with isolated cases being uncommon. Clinically, it can manifest as nonspecific symptoms of the biliary, gastrointestinal, or urinary systems, often mimicking clinical presentations of many digestive diseases, leading to potential misdiagnosis. Genetic sequencing and in-depth embryological research may offer new perspectives for elucidating the etiology and improving diagnostic accuracy. This case highlights the importance of considering gallbladder agenesis in patients presenting with fatty liver, especially in the pediatric population.

## Data Availability

The original contributions presented in the study are included in the article/Supplementary Material, further inquiries can be directed to the corresponding author.
